# Generalising quantum imaginary time evolution to solve linear partial differential equations

**DOI:** 10.1038/s41598-024-70423-5

**Published:** 2024-08-30

**Authors:** Swagat Kumar, Colin Michael Wilmott

**Affiliations:** Department of Physics and Mathematics, Nottingham Trent University, Nottingham, NG11 8NS UK

**Keywords:** Quantum mechanics, Quantum simulation, Qubits, Mathematics and computing, Computational science

## Abstract

The quantum imaginary time evolution (QITE) methodology was developed to overcome a critical issue as regards non-unitarity in the implementation of imaginary time evolution on a quantum computer. QITE has since been used to approximate ground states of various physical systems. In this paper, we demonstrate a practical application of QITE as a quantum numerical solver for linear partial differential equations. Our algorithm takes inspiration from QITE in that the quantum state follows the same normalised trajectory in both algorithms. However, it is our QITE methodology’s ability to track the scale of the state vector over time that allows our algorithm to solve differential equations. We demonstrate our methodology with numerical simulations and use it to solve the heat equation in one and two dimensions using six and ten qubits, respectively.

## Introduction

Quantum computers promise a paradigm shift for computing technology through their capability to solve problems that are inaccessible with classical computers. It is well-understood that classical computers struggle to efficiently solve a class of problems known as optimisation, but a principal promise of quantum computing relates to the significant improvements they bestow on the computational time needed to solve such problems. Quantum computers can be applied to a range of optimisation problems that have widespread value in the real world, including scheduling and planning^1^, biochemical and computational biology^2^, and financial risk^3^. Quantum computers were, however, originally proposed as a means to efficiently simulate quantum Hamiltonian dynamics^4^. Hamiltonian simulation is a task contained in the BQP complexity class^5^, which is significant because tasks in this class are believed to be intractable using classical computers. Many algorithms have been proposed for Hamiltonian simulation^6–11^, and research has continued to improve the technique and performance of such simulations.

Gate-based quantum computers solve the Hamiltonian simulation problem by decomposing the unitary evolution operator into a series of smaller unitaries called quantum gates. The fundamental challenge is how to instruct the quantum computer on the gate set needed to approximate the unitary. Interestingly, the process to undertake Hamiltonian simulation has not yet found an established method of choice. It is achieved by either directly implementing the time evolution operator or by determining the eigenspectrum of the Hamiltonian. State-of-the-art approaches for the former include quantum walks^12^, qubitisation^13^ and quantum signal processing^10^. The latter is an equally difficult approach, and includes the variational quantum eigensolver (VQE)^14–16^ and quantum imaginary time evolution (QITE)^17,18^. These algorithms approximate the ground state of the Hamiltonian and can be applied recursively to obtain the complete eigenspectrum.

Computing the ground state energy of a Hamiltonian is of immense importance, such as in the computation of molecular and material energies^19–21^, and wavefunctions^22–25^. Imaginary time evolution (ITE) is a successful classical method for determining the ground state of a Hamiltonian. By treating time as an imaginary number, the non-unitary time evolution operator generated by ITE does not represent a physical process. Classically solving the imaginary time Schrödinger equation inherits the same computational complexities seen in classical simulations of quantum systems, namely the exponential overhead in maintaining the state of the system. This difficulty inspired the development of QITE as a technique to simulate imaginary time evolution on a quantum computer by evolving a quantum state in imaginary time. In the ideal case, QITE guarantees convergence to the ground state, and, indeed, it is a promising approach as attested by the increasing interest in its potential applications.

As a technique for pursuing the ground state of a Hamiltonian, QITE may be implemented in two ways. Variational QITE^17^ is a hybrid quantum-classical algorithm that considers a system of differential equations linking to gradients of ansatz parameters in imaginary time and coefficients that depend on measurements of the ansatz, both of which contribute to an update rule for finding the ground state. Variational QITE is well suited for noisy intermediate-scale quantum (NISQ) devices as it has a fixed cost ansatz circuit. Conversely, the imaginary time evolution may move out of the ansatz space, implying that convergence may not reflect the true ground state. Furthermore, designing a universal ansatz with a gate count that scales polynomially with the number of qubits is a challenge, explaining why most ansatzes are tailored to the Hamiltonian.

On the other hand, simulated QITE^18^ outlines a quantum approach for simulating imaginary time evolution, by approximating the time evolution operator with Trotter products. This implementation of simulated QITE requires significantly fewer total measurements as compared to the VQE algorithms to achieve the same level of convergence. Simulated QITE with sufficiently large unitary domains does not suffer barren plateaus, as is the case in the variational approach. In contrast, simulated QITE generates circuit depth increases that grow linearly with each imaginary time step.

Research has principally reported on ITE and QITE as methodologies for producing the ground state of a system where, under such implementations, information on the state vector’s direction represents the key ingredient in determining the ground state. More recently, QITE has been used as an approach for solving partial differential equations (PDEs)^26–29^, however, these implementations have been based on variational QITE, which, as mentioned above, may fail to converge if the PDE dynamics move out of the ansatz space. In this paper, we offer an alternative approach for solving linear PDEs that makes use of a simulated QITE implementation. Our extension offers a new avenue for exploration by enlarging the computational reach of the QITE methodology by our algorithm’s ability to track the trajectory and scale of the state vector over time. We demonstrate how to approximate solutions to linear PDEs discretised via finite differences. We also demonstrate our simulated QITE methodology via numerical simulations and use it to solve the heat equation in one and two dimensions.

## Preliminaries

The time evolution of a quantum state, $\psi (\vec {x},t)$, is governed by the Schrödinger equation, which takes the form

1 $$\begin{aligned} i\frac{\partial {\psi (\vec {x},t)}}{\partial t} = \hat{H} {\psi (\vec {x},t)}, \end{aligned}$$

where $\hat{H}$ is a Hermitian linear differential operator known as the Hamiltonian. Since the Hamiltonian is a Hermitian operator, it possesses a spectral decomposition with eigenvalues $\lambda _n$ and corresponding normalised eigenstates ${\psi _n}$. The lowest energy is known as the ground state of the system. Expanding the quantum state $\psi (\vec {x},t)$ at the initial value $t = 0$ in terms of its energy eigenstates, we have it that ${\psi (\vec {x},0)} = \sum _{n}{c_n{\psi _n(\vec {x})}}$, where $c_n$ denotes the overlap of ${\psi (\vec {x},0)}$ and ${\psi _n(\vec {x})}$. The quantum state at a later time *t* is given by ${\psi (\vec {x},t)} = \sum _{n}{c_ne^{-\lambda _nit}{\psi _n}(\vec {x})}$. Applying the variable change $\beta = it$ to Eq. (1) yields the imaginary time Schrödinger equation

2 $$\begin{aligned} \hbar \frac{\partial {\psi (\vec {x},\beta )}}{\partial \beta } = -\hat{H} {\psi (\vec {x},\beta )}. \end{aligned}$$

Since the Hamiltonian $\hat{H}$ remains the same, its solution takes the form ${\psi (\vec {x},\beta =it)} = \sum _{n}{c_ne^{-\lambda _nt} {\psi _n}(\vec {x})}$. The state ${\psi (\vec {x},\beta )}$ represents an exponentially decaying superposition of eigenstates, which, in the limit of $\beta$ large, yields ${\psi (\vec {x},\beta )} = {c_0e^{-\lambda _0\beta } {\psi _0}(\vec {x})}$. This demonstrates that ${\psi (\vec {x},\beta )}$ evolves parallel to the ground state of the system in the limit that imaginary time goes to infinity.

The QITE algorithm simulates the imaginary time evolution of quantum states via the Trotter product approximation. If we express the Hamiltonian as a linear combination of smaller, non-commuting operators $\hat{H} = \sum _{I=1}^{M} \hat{h}_I$, we can approximate the imaginary time evolution operator for a small time step of $\Delta t$ as

3 $$\begin{aligned} e^{-\hat{H}\Delta t} = e^{-\sum _{I=1}^{M}\hat{h}_I\Delta t} = \prod _{I=1}^{M} e^{-\hat{h}_I\Delta t} + O(\Delta t^2). \end{aligned}$$

Since each Trotter step, $e^{-\hat{h}_I\Delta t}$, in the product is non-unitary, the QITE algorithm approximates the normalised action of these operators on a unit quantum state $|\bar{\psi }(t)\rangle$ through a unitary operator $e^{-i\hat{A}\Delta t}$ such that

4 $$\begin{aligned} e^{-i\hat{A}\Delta t}|\bar{\psi }(t)\rangle \approx \frac{e^{-\hat{h}_I\Delta t}|\bar{\psi }(t)\rangle }{\sqrt{\langle \bar{\psi }(t)|e^{-2\hat{h}_I\Delta t}|\bar{\psi }(t)\rangle }}. \end{aligned}$$

The Hermitian operator $\hat{A}$ is expressed as a linear combination of smaller Hermitians, $\hat{A} = \sum _{J=1}^{N} a_J \hat{\sigma }_J$, to ensure that the update can also be expressed as a first-order Trotter product $e^{-i\hat{A}\Delta t} \approx \prod _{J=1}^N e^{-i a_J \hat{\sigma }_J \Delta t}$. Note the choice of $\hat{\sigma }_J$ is such that each term in this product can be efficiently implemented with a parameterised quantum circuit. The coefficients $a_J$ are calculated by solving a system of *N* linear equations constructed using the expectation values $\langle \hat{h}_I\rangle$, $\langle \hat{\sigma }_J^\dagger \hat{\sigma }_{J^\prime }\rangle$ and $\langle \hat{\sigma }_J^\dagger \hat{h}_I\rangle$. Each Trotter step requires taking $O(N^2)$ measurements to construct an $(N \times N)$ matrix equation that generates a circuit of depth *O*(*N*). Overall, simulating $N_T$ time steps with QITE involves taking $O(N_TMN^2)$ measurements.

The support of $\hat{A}$ contains $D=O(C)$ adjacent qubits surrounding the support of $\hat{h}_I$, where *C* denotes the correlation length of the state $|\bar{\psi }\rangle$. For a multi-qubit state, the correlation length *C* is defined to bound the correlations between observables on all pairs of qubits separated by a distance of *L* by $\exp (-L/C)$^18^. Insight into how *C* evolves under the imaginary time evolution allows us to optimise the support of $\hat{A}$ for each Trotter step. However, since *C* is a difficult quantity to compute, we instead use inexact QITE to perform the simulation, assuming a constant domain size *D* which is chosen according to the computational resources available. Increasing values of *D* yield better approximations with the best approximation achieved when *D* equals the total number of interacting qubits. Our numerical implementations are based on inexact QITE for $D=2,4,6$ qubits as the Hamiltonians considered only involve a maximum of six adjacent interacting qubits.

## Simulating PDEs with QITE

QITE was introduced to replicate the imaginary time evolution of an initial state at every time step with the aim of producing the ground state solution. We propose to extend the scope of QITE by reimagining the role of the system’s Hamiltonian $\hat{H}$ beyond its immediate physical interpretation to a differential operator defining a family of linear PDEs spanned by different choices of $\hat{H}$. For instance, by considering the Hamiltonian $\hat{H}$ to be proportional to the Laplace operator $\nabla ^2$, the imaginary time Schrödinger equation can be interpreted as the heat equation given by

5 $$\begin{aligned} \frac{\partial {f(\vec {x},t)}}{\partial t} = \alpha \nabla ^2 {f(\vec {x},t)}. \end{aligned}$$

The heat equation is the quintessential parabolic partial differential equation that has played a fundamental role in developing broader understandings of PDEs. The equation ranks amongst the most widely investigated topics in the physical sciences. The heat equation bridges to probability theory through its connection with the study of random walks and Brownian motion via the Fokker–Planck equation^30^. The Black–Scholes equation^31^ of financial mathematics can be seen as a variant of the heat equation, and the Schrödinger equation reduces to a heat equation in imaginary time. From this position, QITE, offers an appealing route for simulating the normalised dynamics of this family of PDEs.

QITE seeks to determine the ground state of the system, where information relevant to the solution state is extracted from the state by taking measurement on the final quantum state. In practice, it should be expected that the number of distinct measurements required to extract this relevant information will scale polynomially with the number of qubits. For instance, in the case of the natural sciences, we typically observe associated Hamiltonians having a polynomial number of non-zero terms in the Pauli basis. Determining the exact quantum state requires quantum state tomography and exponentially many measurements. However, if we restrict ourselves to simulating non-negative functions, we can reconstruct the quantum state using only the probability distribution of the Pauli Z, computational basis, measurements, since the amplitudes of the quantum state are the square roots of the measurement probabilities of each computational basis state. For this reason, we will simulate the heat equation in one and two dimensions for non-negative solutions. To achieve this, and establish QITE as a methodology for simulating PDEs, we require, firstly, to discretise the system and, secondly, to encode the Hamiltonian in the Pauli basis.

### Discretising space

Propagation of a quantum state as determined by the Schrödinger equation, Eq. (1), is defined on a domain of continuous space. To simulate these dynamics with a discrete set of qubits, we are required to discretise the continuous wavefunction $\psi (\vec {x},t)$ to a discrete qubit state vector $|\bar{\psi }(t)\rangle$, and calculate the corresponding qubit Hamiltonian. We encode the continuous linear differential operator to a finite difference matrix. We will first consider the one dimensional case before generalising to higher dimensions.

#### One dimensional space

Let us consider a function defined on a one dimensional space domain $f:[a,b) \rightarrow \mathbb {C}$. This function can be encoded into the state vector of *n*-qubits by storing $N=2^n$ uniformly spaced samples of the function in an unnormalised state vector

6 $$\begin{aligned} |f\rangle = \sum _{k=0}^{N-1} f\left( a + kh\right) |k\rangle = \sum _{k=0}^{N-1} f_k |k\rangle , \end{aligned}$$

with the spacing $h = \frac{b-a}{N}$ and $|k\rangle$ denoting elements of standard basis. Next, let us consider approximating a linear partial differential operator on the discretised space using the method of finite difference approximation of derivatives. A first order finite difference takes the form $f(x+b)-f(x+a)$ and is classified as the central difference when we have $\delta ^1_h[f](x) = f(x+h/2) - f(x-h/2)$, for spacing *h*. Higher order partial differential operators are approximated by the central finite differences given by

7 $$\begin{aligned} \frac{\partial ^m f(x)}{\partial x^m} \approx \frac{\delta _h^m[f] (x)}{h^m} \ \text {where} \ \delta _h^m[f] (x) = \sum _{i=0}^m (-1)^i \left( {\begin{array}{c}m\\ i\end{array}}\right) f\left( x + \left( \frac{m}{2} - i\right) h\right) . \end{aligned}$$

Of particular interest is the second order partial differential operator that appears in the heat equation, which can be approximated by the difference operator

8 $$\begin{aligned} \hat{\delta }_{h}^{2} |f\rangle = \frac{1}{h^2} \sum _{k=0}^{N-1} (f_{k+1} - 2f_k + f_{k-1}) |k\rangle . \end{aligned}$$

The boundary conditions determine the values of $f_{-1}=f(a-h)$ and $f_N=f(b)$. The difference operator under the zero boundary conditions, $f_{-1} = f_N = 0$, is represented by the following matrix written in the standard basis

9 $$\begin{aligned} \frac{1}{h^2} \begin{pmatrix} -2 &  1 &  &  &  \\ 1 &  -2 &  1 &  &  \\ &  1 &  \ddots &  \ddots &  \\ &  &  \ddots &  \ddots &  1 \\ &  &  &  1 &  -2 \end{pmatrix}. \end{aligned}$$

The difference operator under periodic boundary conditions, $f_{-1}=f_{N-1}$ and $f_N=f_0$, is represented by the following matrix written in the standard basis

10 $$\begin{aligned} \frac{1}{h^2} \begin{pmatrix} -2 &  1 &  &  &  1\\ 1 &  -2 &  1 &  &  \\ &  1 &  \ddots &  \ddots &  \\ &  &  \ddots &  \ddots &  1 \\ 1 &  &  &  1 &  -2 \end{pmatrix}. \end{aligned}$$

Let $\hat{D}^{(n)}_0$ denote the *n*-qubit second-order finite difference Hamiltonian under zero boundary conditions such that $\hat{D}^{(n)}_0 = h^2 \hat{\delta }_{h}^{2}.$ It then follows that, in the Pauli operator basis,

11 $$\begin{aligned} \hat{D}_0^{(1)} = \begin{pmatrix} -2 &  1 \\ 1 &  -2 \end{pmatrix} = {-2\hat{I} + \hat{X}} . \end{aligned}$$

To determine the Pauli basis representation of *n*-qubit second-order finite difference Hamiltonian under zero boundary conditions, we define

12 $$\begin{aligned} \hat{A}^{(n)}_{\swarrow }:= \hat{A}^{(1)}_{\swarrow } \otimes \hat{A}^{(n-1)}_{\swarrow } \quad \text{where} \quad \hat{A}^{(1)}_{\swarrow }:= \frac{\hat{X} - i\hat{Y}}{2} = \begin{pmatrix} 0 &  0 \\ 1 &  0 \end{pmatrix}, \end{aligned}$$

and

13 $$\begin{aligned} \hat{A}^{(n)}_{\nearrow }:= \hat{A}^{(1)}_{\nearrow } \otimes \hat{A}^{(n-1)}_{\nearrow }\quad \text{where} \quad \hat{A}^{(1)}_{\nearrow }:= \frac{\hat{X} + i\hat{Y}}{2} = \begin{pmatrix} 0 &  1 \\ 0 &  0 \end{pmatrix}. \end{aligned}$$

The two-qubit Hamiltonian is given as

14 $$\begin{aligned} \hat{D}^{(2)}_0 = \left( \begin{array}{cc|cc} -2 &  1 &  0 &  0 \\ 1 &  -2 &  1 &  0 \\ \hline 0 &  1 & -2 &  1 \\ 0 &  0 &  1 & -2 \end{array}\right) . \end{aligned}$$

Using Eqs. (11)–(13), we have

15 $$\begin{aligned} \hat{D}^{(2)}_0 = \begin{pmatrix} \hat{D}^{(1)}_0 &  \hat{A}^{(1)}_{\swarrow } \\ \hat{A}^{(1)}_{\nearrow } &  \hat{D}^{(1)}_0 \end{pmatrix} = \hat{I} \otimes \hat{D}^{(1)}_0 + \hat{A}^{(1)}_{\swarrow } \otimes \hat{A}^{(1)}_{\nearrow } + \hat{A}^{(1)}_{\nearrow } \otimes \hat{A}^{(1)}_{\swarrow }. \end{aligned}$$

From Eq. (9), we can show that the *n*-qubit Hamiltonian $\hat{D}^{(n)}_0$ has the form

16 $$\begin{aligned} \hat{D}^{(n)}_0 = \begin{pmatrix} \hat{D}^{(n-1)}_0 &  \hat{A}^{(n-1)}_{\swarrow } \\ \hat{A}^{(n-1)}_{\nearrow } &  \hat{D}^{(n-1)}_0 \end{pmatrix} = \hat{I} \otimes \hat{D}^{(n-1)}_0 + {\hat{A}^{(1)}_{\swarrow }} \otimes \hat{A}^{(n-1)}_{\nearrow } + {\hat{A}^{(1)}_{\nearrow }} \otimes \hat{A}^{(n-1)}_{\swarrow }. \end{aligned}$$

Similarly, we define $\hat{D}^{(n)}_p$ to be the *n*-qubit second-order finite difference Hamiltonian under periodic boundary conditions. It can be shown from Eq. (10) that $\hat{D}^{(n)}_p$ takes the form

17 $$\begin{aligned} \hat{D}^{(n)}_p = \hat{D}^{(n)}_0 + \hat{A}^{(n)}_{\swarrow } + \hat{A}^{(n)}_{\nearrow }. \end{aligned}$$

We note that the number of Pauli strings in this decomposition scales exponentially with the number of qubits, $M = O(2^n)$. On the other hand, the number of terms involving tensor product strings of $\hat{A}_\swarrow$ and $\hat{A}_\nearrow$ grows linearly, $M = O(n)$. Interestingly, we have become aware of a protocol to measure the expectation values, $\langle \hat{h}_I\rangle$, expressed in terms of these tensor products with a single ancilla qubit^32^, allowing an exponential reduction in the time complexity of this method.

#### Higher dimensional space

Generalising the state encoding for the one dimensional case to higher space dimensions is achieved by taking the tensor product of the qubit registers of the associated dimensions. For example, a function defined on a two-dimensional space domain $f : [a_1,b_1)\times [a_2,b_2) \rightarrow \mathbb {C}$ can be encoded with 2*n* qubits by storing $N^2$ samples in the unnormalised state vector

18 $$\begin{aligned} |f\rangle = \sum _{k_1, k_2 = 0}^{N-1} f(a_1 + k_1h_1,\ a_2 + k_2 h_2) |k_1\rangle |k_2\rangle = \sum _{k_1, k_2=0}^{N-1} f_{k_1,\ k_2} |k_1\rangle |k_2\rangle , \end{aligned}$$

with spacings $h_i = \frac{b_i - a_i}{N}$ for $i=1,2$. Similarly, we can construct the associated finite difference operator by taking the tensor products of the underlying one dimensional operators. For instance, the two dimensional Laplace operator, $\nabla ^2 = \partial ^2_x + \partial ^2_y$, is represented by the following 2*n*-qubit finite difference operator

19 $$\begin{aligned} \hat{L}^{(n)}_{h_1, h_2}|f\rangle =&\left[ \frac{\hat{D}^{(n)}}{h_1^2} \otimes \hat{I}^{\otimes n} + \hat{I}^{\otimes n} \otimes \frac{\hat{D}^{(n)}}{h_2^2}\right] |f\rangle \nonumber \\ =&\sum _{k_1,k_2 = 0}^{N-1} \left( \frac{f_{k_1+1,\ k_2} - 2f_{k_1,\ k_2} + f_{k_1-1,\ k_2}}{h_1^2} + \frac{f_{k_1,\ k_2+1} - 2f_{k_1,\ k_2} + f_{k_1,\ k_2-1}}{h_2^2}\right) |k_1\rangle |k_2\rangle . \end{aligned}$$

The Laplace operator, acting as the Hamiltonian to the Heat equation, contains interactions of successive samples in each spatial dimension. Under the discretisation scheme defined in Eq. (8), these interactions imply that the correlation length is bounded linearly with the number of qubits per dimension.

### Obtaining solutions from the state vector

Although QITE simulates the trajectory of the PDE solution, it does not account for how the length of the state vector changes over time. To achieve our intended application, we must also approximate the norm at each time step and rescale the state vectors obtained from QITE to match the complete dynamics of the PDE solution.

#### Measuring the state vector

If we know that the original function only takes on non-negative values in the region we are solving for, the state vector $|f\rangle$ will only have non-negative amplitudes in the computational basis. We will, therefore, restrict ourselves to PDEs involving only even-ordered differential operators as they are Hermitian and represented by real matrices in the computational basis. This ensures that the quantum state $|f\rangle$ will not contain any phase information and can be reconstructed by taking the square root of its computational basis measurement probability distribution.

#### Reconstructing the norm

In the reconstruction of the norm, we seek to approximate the squared norm, $c(k\Delta t) = \langle \bar{\psi }((k-1)\Delta t)|e^{-2\hat{H}\Delta t}|\bar{\psi }((k-1)\Delta t)\rangle$, of the non-unitary evolution operator $e^{-\hat{H}\Delta t}$ at each simulated time step of size $\Delta t$. To account for how QITE simulates each time step with *M* Trotter steps, we define the normalised state produced by QITE after $\kappa$ Trotter steps as $|\bar{\psi }(\frac{\kappa }{M}\Delta t)\rangle .$ QITE approximates *c*(*t*) by taking the product of the linear order approximations of the squared norm obtained in each Trotter step,

20 $$\begin{aligned} c^\prime ((k+1)\Delta t) = \prod _{I=1}^M \left[ 1 - 2\Delta t \left\langle {\bar{\psi }\left( k\Delta t+\frac{I-1}{M}\Delta t\right) }\right| \hat{h}_I \left| {\bar{\psi }\left( k\Delta t+\frac{I-1}{M}\Delta t\right) }\right\rangle \right] . \end{aligned}$$

Assuming that a QITE implementation of $N_T$ time steps has perfect fidelity, we can express the vector containing samples of the PDE solution, $f(x,t=N_T\Delta t)$, as

21 $$\begin{aligned} |f(N_T\Delta t)\rangle = \Vert |f(0)\rangle \Vert \left( \prod _{k=1}^{N_T-1} \sqrt{c(k\Delta t)}\right) |\bar{\psi }(N_T\Delta t)\rangle . \end{aligned}$$

The theoretical squared norm at the *k*-th time step is given as the product of the *k* individual squared norms

22 $$\begin{aligned} C_f((k+1)\Delta t) = \prod _{j=1}^{k} c(j\Delta t). \end{aligned}$$

An approach to approximate $C_f(t)$ would be to consider the product of the linear approximants

23 $$\begin{aligned} C^\prime ((k+1)\Delta t) = \prod _{j=1}^{k} c^\prime (j\Delta t). \end{aligned}$$

The issue with this approach is that the relative errors associated to $c^\prime (j\Delta t)$, for $j =1,\ldots ,k$, compound in the product, which leads to a significant deviation from the theoretical norm with each additional time step. To mitigate the accumulation of errors in the running product, $C^\prime (k\Delta t)$, we undertake a strategy to rescale the norm after every *K* time steps. To implement this strategy, we require the normalised ground state of the Hamiltonian, $|\bar{\psi }_0\rangle$, and its associated eigenvalue, $\lambda _0$. Let $C_*(t)$ denote a good approximation for the squared norm. Under the assumption that our QITE simulation has high fidelity, that is, ${|f(t)\rangle \approx \Vert |f(0)\rangle \Vert \sqrt{C_*(t)} |\bar{\psi }(t)\rangle }$, we then have it that

24 $$\begin{aligned} {\frac{\langle \bar{\psi }_0|f(t)\rangle }{\Vert |f(0)\rangle \Vert }} \approx \sqrt{C_*(t)}{\langle \bar{\psi }_0|\bar{\psi }(t)\rangle }. \end{aligned}$$

Using $|f(t)\rangle = \sum _{l=0}^{N-1}{e^{-\lambda _l t}} |\bar{\psi }_l\rangle \langle \bar{\psi }_l||f(0)\rangle$, it follows that

25 $$\begin{aligned} \begin{aligned} {\frac{\langle \bar{\psi }_0|f(t)\rangle }{\Vert |f(0)\rangle \Vert }}&= \frac{\langle \bar{\psi }_0|\left( \sum _{l=0}^{N-1}{e^{-\lambda _l t}} |\bar{\psi }_l\rangle \langle \bar{\psi }_l|\right) |f(0)\rangle }{\Vert |f(0)\rangle \Vert } \\&= \sum _{l=0}^{N-1}{e^{-\lambda _l t}}\langle \bar{\psi }_0| \bar{\psi }_l\rangle \langle \bar{\psi }_l|\frac{|f(0)\rangle }{\Vert |f(0)\rangle \Vert } \\&= \sum _{l=0}^{N-1}{e^{-\lambda _l t}}\delta _{l,0}\langle \bar{\psi }_l|\frac{|f(0)\rangle }{\Vert |f(0)\rangle \Vert } \\&= e^{-\lambda _0 t}\langle \bar{\psi }_0|\bar{f}(0)\rangle , \end{aligned} \end{aligned}$$

and, consequently, Eq. (24) may be rewritten as

26 $$\begin{aligned} e^{-\lambda _0 t}{\langle \bar{\psi }_0|\bar{f}(0)\rangle } \approx {\sqrt{C_*(t)} \langle \bar{\psi }_0|\bar{\psi }(t)\rangle }, \end{aligned}$$

from which we deduce an approximation for $C_*(t)$ as

27 $$\begin{aligned} C_*(t) \approx e^{-2\lambda _0 t}\frac{\langle \bar{\psi }_0|\bar{f}(0)\rangle ^2}{\langle \bar{\psi }_0|\bar{\psi }(t)\rangle ^2} . \end{aligned}$$

Note that calculating $C_*$ requires a measuring the state vector $|\bar{\psi }(t)\rangle$. After every *K* time steps, we measure the state vector and rescale our norm approximation to $C_*$, giving us an improved approximation of the squared norm;

28 $$\begin{aligned} C_\psi (k\Delta t) = {\left\{ \begin{array}{ll} C_*(k\Delta t) &  \text {for } k \equiv 0\ \text {mod}\ K \\ C_\psi ((k-1)\Delta t) \cdot c^\prime (k\Delta t) &  \text {for } k \not \equiv 0\ \text {mod}\ K \end{array}\right. }. \end{aligned}$$

**Figure 1:**
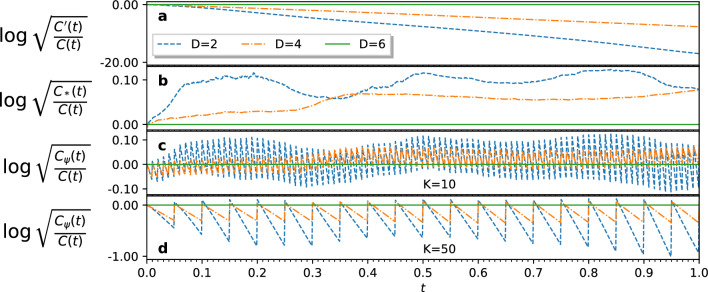
Comparison of different norm reconstruction strategies. The graphs show the base-10 logarithms of the ratios of the reconstructed norms to the analytical norm for a 6-qubit square wave evolution against the heat equation with $\alpha =0.8$ over 1000 simulated time steps. **(a)** We see how the errors in $c^\prime$ compound in the product $C^\prime$. **(b)** The fidelity of our QITE simulation increases with *D*, and we see how $C_*$ is a better approximation for the norm at higher fidelities. **(c)** $C_\psi$ combines information from $c^\prime$ and $C_*$, and is, on average, a better approximation of the norm for sufficiently small *K*. **(d)** At higher values of *K*, the errors in $c^\prime$ are able to compound, making the approximation worse on average.

### Comparing QITE with analytical evolution

Our methodology performs a unitary approximation of a linear PDE and provides an estimate on how the norm evolves. This information allows us to obtain approximate solutions to the PDE. Let $|\bar{f}(t)\rangle = |f(t)\rangle /\sqrt{C_f(t)}$ denote the normalised state vector containing scaled samples of the analytical solution *f*(*x*, *t*) of the PDE, and $|{\psi }(t)\rangle = \sqrt{C_\psi (t)} |\bar{\psi }(t)\rangle$ denote the rescaled state vector containing samples of the solution $\psi (x,t)$ approximated by our QITE methodology. When restricted to functions that only take on non-negative values, the fidelity of our QITE implementation, given by

29 $$\begin{aligned} F(t) = \langle \bar{f}(t)|\bar{\psi }(t)\rangle , \end{aligned}$$

measures the accuracy of our normalised evolution. The ratio between the approximated and analytical norms is

30 $$\begin{aligned} r(t) = \sqrt{\frac{C_\psi (t)}{C_f(t)}}, \end{aligned}$$

which measures the accuracy of our reconstruction. For *N* samples, we can write the mean squared error, MSE, as

31 $$\begin{aligned} \begin{aligned} \text {MSE}(t)&= \frac{\Vert |f(t)\rangle - |\psi (t)\rangle \Vert ^2}{N}\\&= \frac{C_f(t) + C_\psi (t) - 2\sqrt{C_f(t) C_\psi (t)}\langle \bar{f}(t)|\bar{\psi }(t)\rangle }{N} \\&= \frac{C_f(t) + r^2(t) C_f(t) - 2 r(t) C_f(t) F(t)}{N} \\&= \frac{2C_f(t)}{N}\left[ \frac{1+r^2(t)}{2} - r(t)F(t)\right] . \end{aligned} \end{aligned}$$

Equation (31) demonstrates the mean squared error to be a useful metric because it correlates to both the fidelity and norm ratio of our approximation.

## Results

To demonstrate how our QITE methodology can be used to solve PDEs, we target a simulation of the heat equation, Eq. (5). When expressed in terms of the imaginary time Schrödinger equation, the Hamiltonian operator corresponding to the heat equation is $\hat{H} = -\alpha \nabla ^2$. We performed numerical simulations of our QITE methodology for domain sizes $D=2,4$ and 6, from which we obtained approximate solutions to the heat equation for various initial states and boundary conditions. Across our experiments, we set $\alpha =0.8$, simulated the dynamics from $t=0$ to $t=1$, and used a grid spacing of $h=0.1$, and a time step $\Delta t=0.001$. We chose these values for $\Delta t$ and *h* to satisfy the von Neumann stability criteria for the forward time central space methods for solving the heat equation^33,34^. The results reported in Fig. 1 allowed us to decide on a norm correction frequency of $K=10$, as the log norm ratio oscillated around zero for this choice of constants.

**Figure 2:**
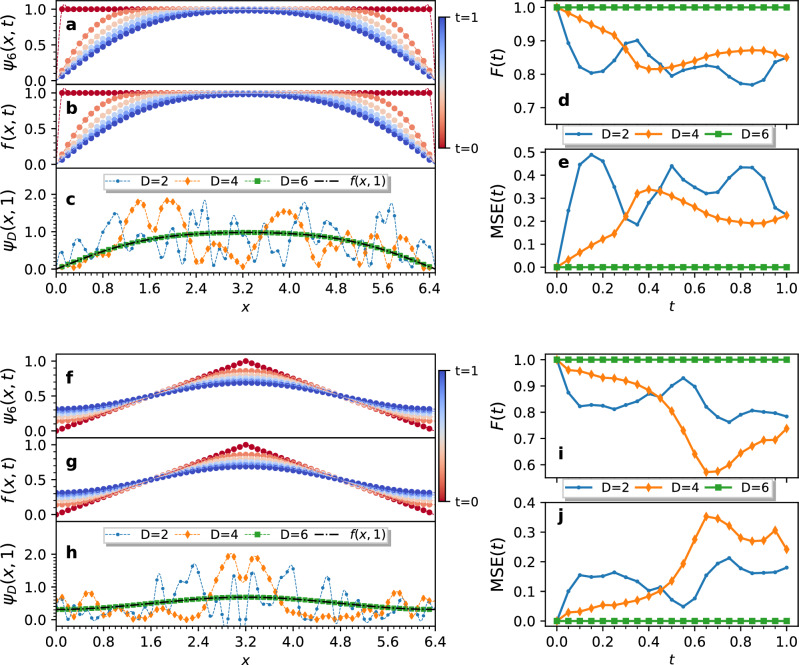
QITE simulations for the heat equation in one spatial dimension. The figure compares our QITE solutions for various domain sizes *D*, $\psi _D(x,t)$, to the analytical solutions, *f*(*x*, *t*), of the heat equation with $\alpha =0.8$. The function samples were encoded in the state vector of 6 qubits. We show results of two simulations; **(a–e)** refer to a square wave with zero boundary conditions, while **(f–j)** refer to a triangle wave with periodic boundary conditions. The norm was corrected at every $K=10$ simulated time steps. **(a,f)** Show the solutions obtained from QITE for a domain size of $D=6$ at different times. **(b,g)** Show the analytical solutions *f*(*x*, *t*) at the same time steps, as indicated by the color of the curves. The dots indicate the function samples, connected by their Fourier interpolations. **(c,h)** Compare the states produced by the $D=2,4,6$ QITE approximations at time $t=1$ to the corresponding analytical solution. Using inexact QITE, the convergence of the algorithm improves with larger domain sizes *D*. **(d,i)** Show the fidelity of the QITE approximations over time. **(e,j)** Show the mean squared error of the QITE approximations to the analytical solutions over time.

**Figure 3:**
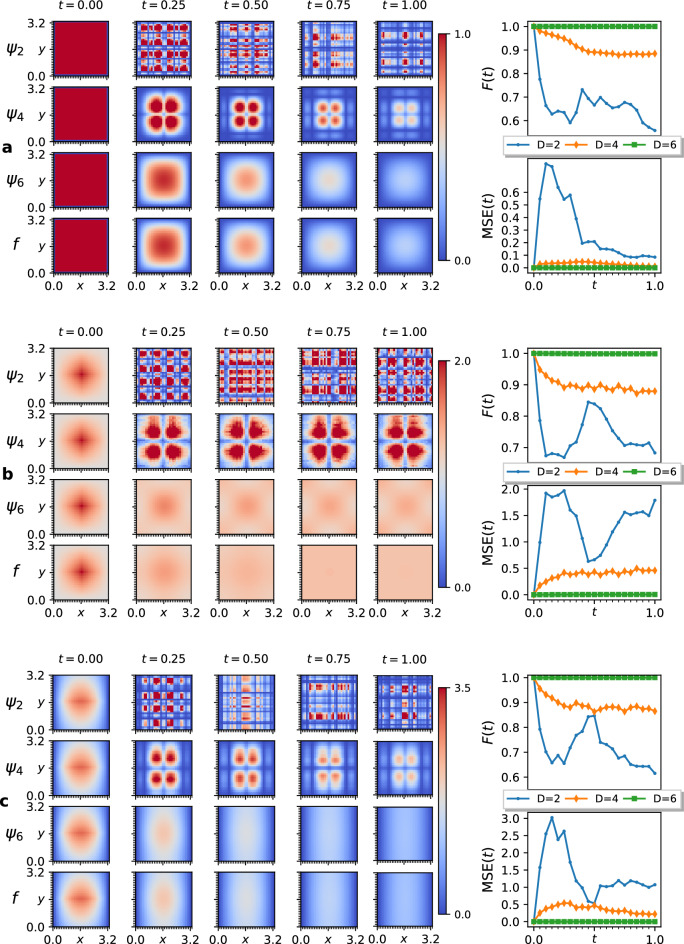
QITE simulations for the heat equation in two spatial dimensions. The figure compares our QITE solutions with domain size *D*, $\psi _D(x,y,t)$, to the analytical solutions, *f*(*x*, *y*, *t*), of the two-dimensional heat equation with diffusion coefficient $\alpha =0.8$. The function samples were encoded in the state vector of 10 qubits, with the *x* and *y* axes mapped to 5 qubits each. The norm was corrected at every $K=10$ time steps. The plots on the left show the QITE solutions corresponding to $D=2,4,6$ and the analytical solution (top to bottom) at different times (columns), with the colour of pixels corresponding to the value of the function according to the color bar. The fidelity and mean squared error of the approximations are also displayed on the right. The initial states and boundary conditions for the experiments are as follows: in **(a)**, the initial state is a two-dimensional square wave with zero boundary conditions in both *x* and *y* directions; in **(b)**, the initial state is a product of triangle waves in *x* and *y* with height 1, a total offset of 1, and periodic boundary conditions in both *x* and *y* directions; in **(c)**, the initial state is a product of a triangle wave in *y* with height 1, and an inverted parabola in *x* with a maximum height of 1.5, and zero boundary conditions in the *x* direction and periodic boundary conditions in the *y* direction.

Our implementation of the QITE methodology as a PDE solver supports two families of boundary conditions, namely, the zero boundary conditions, $f(0)=f(L)=0$, and periodic boundary conditions, $f(x)=f(x+L)$. Figure 2 demonstrates the results of our simulations for the one dimensional heat equation for the zero and periodic boundary conditions. We stored the solutions in the state vector of $n=6$ qubits, giving us the boundary lengths $L=6.5$ in the case of the zero boundary conditions, and $L=6.4$ in the case of the periodic boundary conditions. Since the $D=6$ approximation covers the entire set of interacting qubits, our QITE implementation demonstrated a perfect fidelity to the analytical solution and zero mean squared error. Figure 3 demonstrates our solutions for the two-dimensional heat equation, where we considered all combinations of zero and periodic boundary conditions in each spatial dimension. We used 10 qubits to store the function samples, distributing $n=5$ sampling qubits for both *x* and *y* directions, which yielded boundary lengths $L=3.3$ for the zero boundary conditions, and $L=3.2$ for periodic boundary conditions. Since the two-dimensional Laplace Hamiltonian, shown in Eq. (19), does not have interactions between the *x* and *y* axes’ sampling qubits, we chose unitary domains to cover the five *x* and *y* qubits individually. This allowed the $D=6$ approximation to cover the entire set of interacting qubits, again yielding perfect fidelity to the analytical solutions and zero mean squared error.

## Discussion

As regards to the norm correction frequency, we empirically determined that a norm correction frequency $K=10$ was sufficient to approximate the dynamics of the heat equation for our choice of constants. Further investigation is needed to determine a logical correlation between the choice of constants and a suitable value for *K*. In the case of when norm correction is required but that the exact ground state is not known, we can estimate this state by running a QITE simulation over a long period of imaginary time to get approximations for the ground state, $|\bar{\Psi }_0\rangle \approx |\bar{\psi }_0\rangle$ and its eigenvalue, $\Lambda _0 = \langle \bar{\Psi }_0|\hat{H}|\bar{\Psi }_0\rangle \approx \lambda _0$. Adopting this state as a heuristic for the ground state, we can substitute $\Lambda _0$ and $|\bar{\Psi }_0\rangle$ in Eq. (27) to get an approximate norm correction factor. Note that this approximation does not affect the fidelity of the solutions. In relation to the function encoding schemes, the finite difference matrices approximating the second derivative operator have at most three non-zero entries in each row. These entries indicate the interaction of the basis states as they pertain to the determination of the basis state amplitudes in the final state vector. In particular, the output amplitude of basis state $|k\rangle$ depends on the basis states $|k-1\rangle , |k\rangle ,$ and $|k+1\rangle$. Under our encoding scheme, each basis state $|k\rangle$ is mapped to elements of the computational basis state in lexicographical order. Consequently, under the direct encoding scheme, approximations for $D<n$ are unable to capture all the interactions in the finite difference matrix. This is most easily seen in the horizontal and vertical bands in the centre of the $D=4$ approximations in Fig. 3. A different encoding scheme that that reduces the correlation length of the quantum states under the finite difference Hamiltonian may allow us to capture all possible qubit interactions for $D<n$ and should permit perfect fidelity that benefits with improvements compared to the direct encoding above. Our intention is to consider this issue as a basis for future work. Finally, as regards to general boundary conditions, the QITE methodology examined here only allows us to capture the zero and periodic boundary conditions, since these conditions correspond to Hermitian finite difference matrices.

## Conclusions

In this work, we demonstrated a new and practical application of QITE as a quantum numerical solver for linear PDEs. Our methodology adopts QITE’s ability to model the normalised trajectory of a quantum state. Additionally, our methodology also tracks the scale of the state vector over time. It is the interaction between these two features that has enabled us to broaden the scope of QITE to approximate solutions to linear PDEs discretised via finite differences. Using numerical simulations, we implemented our methodology to solve the heat equation in one and two dimensions, using six and ten qubits, respectively. In our experiments, we demonstrated perfect fidelity along with a mean squared error converging to zero.

### Supplementary Information

Supplementary Information.

## Data Availability

All data generated and analysed during this study are included in this published article and its Supplementary Information files.

## Code availability

The code that supports the findings of this study is available from the corresponding authors upon reasonable request.
